# Applying a complex adaptive systems approach to the evaluation of a school-based intervention for intimate partner violence prevention in Mexico

**DOI:** 10.1093/heapol/czaa067

**Published:** 2020-08-06

**Authors:** Shelly Makleff, Marissa Billowitz, Jovita Garduño, Mariana Cruz, Vanessa Ivon Silva Márquez, Cicely Marston

**Affiliations:** c1 Faculty of Public Health and Policy, London School of Hygiene and Tropical Medicine, 15-17 Tavistock Place, London WC1H 9SH, UK; c2 Independent, Juárez 208, Col. Tlalpan Centro, 14000 Mexico City, Mexico; c3 Fundación Mexicana para la Planeación Familiar, A.C. (Mexfam), Juárez 208, Col. Tlalpan Centro, 14000 Mexico City, Mexico; c4 IPPF/WHR Mexico, Juárez 208, Col. Tlalpan Centro, 14000 Mexico City, Mexico

**Keywords:** Complex adaptive systems, evaluation of complex interventions, intimate partner violence, complexity, implementation research, gender-transformative approach, comprehensive sexuality education

## Abstract

Despite calls for evaluation practice to take a complex systems approach, there are few examples of how to incorporate complexity into real-life evaluations. This article presents the case for using a complex systems approach to evaluate a school-based intimate partner violence-prevention intervention. We conducted a *post hoc* analysis of qualitative evaluation data to examine the intervention as a potential system disruptor. We analysed data in relation to complexity concepts particularly relevant to schools: ‘diverse and dynamic agents’, ‘interaction’, ‘unpredictability’, ‘emergence’ and ‘context dependency’. The data—two focus groups with facilitators and 33 repeat interviews with 14–17-year-old students—came from an evaluation of a comprehensive sexuality education intervention in Mexico City, which serves as a case study for this analysis. The findings demonstrate an application of complex adaptive systems concepts to qualitative evaluation data. We provide examples of how this approach can shed light on the ways in which interpersonal interactions, group dynamics, the core messages of the course and context influenced the implementation and outcomes of this intervention. This gender-transformative intervention appeared to disrupt pervasive gender norms and reshape beliefs about how to engage in relationships. An intervention comprises multiple dynamic and interacting elements, all of which are unlikely to be consistent across implementation settings. Applying complexity concepts to our analysis added value by helping reframe implementation-related data to focus on how the ‘social’ aspects of complexity influenced the intervention. Without examining both individual and group processes, evaluations may miss key insights about how the intervention generates change, for whom, and how it interacts with its context. A social complex adaptive systems approach is well-suited to the evaluation of gender-transformative interventions and can help identify how such interventions disrupt the complex social systems in which they are implemented to address intractable societal problems.

**Key Messages**

- A social complex adaptive systems approach is well-suited to the evaluation of gender-transformative interventions and can help identify how such interventions disrupt the complex social systems in which they are implemented to address intractable societal problems, such as intimate partner violence.
- Without examining both individual and group processes, evaluations may miss key insights about how the intervention generates change, for whom, and how it interacts with its context. A complex adaptive systems approach can complement individual-level analyses to provide information about observed variability in implementation environments, actors and outcomes.
- Even interventions implemented with high fidelity in nearly identical conditions will almost certainly manifest differently between individuals and groups. This does not necessarily reflect implementation success or failure but rather can be expected due to the nature of social interventions as embedded in complex systems—which are context dependent, behave unpredictably and have emergent results that develop over time.
- From a programme design and implementation perspective, systems analysis can help organizations prepare for different types of unpredictable occurrences and outcomes that may emerge during an intervention by focusing on learning from the most challenging aspects of implementation. Well-trained facilitators and a flexible curriculum can help implementing teams cope with unpredictability and suboptimal implementation conditions.

## Introduction

Despite calls over the last decade for evaluation practice to take a complex systems approach and move beyond the individual to explore macro-level effects, there are few examples of how to incorporate the concept of complexity into real-life evaluations (Smith and Petticrew, 2010; Craig and Petticrew, 2013; Moore *et al.*, 2019). Interest in evaluating complex and social interventions has grown steadily (Craig and Petticrew, 2013), yet ‘the literature […] is thick with descriptions of complex, challenging interventions, but thin on practical advice on how these should be dealt with’ (Datta and Petticrew, 2013). Reviews have found that clinical and health promotion interventions, and hospitals and schools, are commonly the subject and sites of research discussing complexity (Datta and Petticrew, 2013; Thompson *et al.*, 2016). However, applications of complexity theory for interventions that address complex phenomena driven by underlying social norms, e.g. intimate partner violence (IPV), are rare. One study in New Zealand applied complexity theory to conceptualize the healthcare system response to IPV but did not mention the additional complexities of social norms or gender (Gear *et al.*, 2018). Taking these into account is important, as social norms are important drivers of the harmful global phenomenon of IPV (Jewkes *et al.*, 2019) and gender itself is a complex social system that defines what we expect of women and men in any given society (Hirdman, 1991; Heise *et al.*, 2019). There is a growing evidence base examining community-based interventions that address gendered social norms as part of IPV prevention efforts (Jewkes *et al.*, 2019); however, these studies rarely adopt a complex systems approach and few are carried out in schools. Here, we consider the case for using a complex adaptive systems framework to evaluate school-based IPV prevention interventions.

Complexity, often considered in the evaluation literature to be an attribute of an intervention, can alternatively be conceived of as a characteristic of the system or setting in which an intervention takes place (Shiell *et al.*, 2008; Hawe, 2015). Building on this, Moore *et al.* (2019) conceptualize interventions as events that aim to disrupt complex systems. This moves the focus of evaluative research away from individual behavioural change (Westhorp, 2012) to instead examining how a complex system—such as a hospital, school or community—responds to an intervention over time (Moore *et al.*, 2019). Evaluation conducted with a complex systems approach can seek to understand how an intervention—an event in a system—‘begins to gain traction within its context over time’ to ‘either leave a lasting footprint or wash out’ (Hawe *et al.*, 2009). Context is a necessary focus when implementing or evaluating interventions with a complex systems perspective, as the ‘effects of any intervention are influenced strongly by the starting points of the system they attempt to disrupt’ (Moore *et al.*, 2019). In other words, the type and extent of change that an intervention creates in a system reflects local or particular characteristics.

Beyond the properties of the system in which an intervention is implemented, additional complexities may reflect the types of outcomes being addressed. IPV is a complex social phenomenon, and prevention programming may be more effective when it intervenes at multiple levels beyond the individual, e.g. the relationship, community and societal levels (Heise *et al.*, 1999; Morrison *et al.*, 2004; Meinck *et al.*, 2019). IPV prevention interventions often use a ‘gender-transformative’ approach, which aims to shift gendered power differentials to become more equal (Dworkin *et al.*, 2015; Jewkes *et al.*, 2015; Michau *et al.*, 2015; Jewkes *et al.*, 2019; Ruane-McAteer *et al.*, 2019). Such shifts, which are central to gender-transformative programmes, are also complex (Walters, 2004). IPV prevention interventions are usually implemented in group settings, such as communities, schools or families (Garcia-Moreno *et al.*, 2014; Lundgren and Amin, 2015; Jewkes *et al.*, 2019). We adopt the conceptualization of schools—and other group settings—as ‘social’ complex adaptive systems (Keshavarz *et al.*, 2010), which comprise ‘a collection of individual agents with freedom to act in ways that are not always totally predictable, and whose actions are interconnected so that one agent’s actions changes the context for other agents’ (Plsek and Greenhalgh, 2001). Given the complexities inherent to IPV programming, a social complex adaptive systems approach may be well-suited for evaluating prevention interventions.

We present an illustrative case study applying a complex adaptive systems approach to an evaluation of a school-based comprehensive sexuality education intervention with a focus on preventing IPV. The study objectives are to consider whether complex adaptive systems concepts appear relevant for the evaluation; how this approach helps understand the intervention; and what it may add beyond a traditional evaluation perspective. We also aim to identify ‘disruptive’ elements of the intervention and examine how group dynamics and social context can influence participant experiences and intervention outcomes.

## Methods

### Theoretical perspective

Schools have many of the key characteristics of ‘social’ complex adaptive systems—they comprise a population of diverse and changing individuals (or ‘agents’) such as students and teachers, who interact in often unpredictable ways that are linked to context, leading to non-linear and emergent outcomes (Keshavarz *et al.*, 2010). Therefore, a complex systems lens is appropriate for research about school-based interventions (Keshavarz *et al.*, 2010; Moore *et al.*, 2019). Keshavarz *et al.* (2010) identified key attributes of complex adaptive systems as particularly relevant to schools: ‘diverse and dynamic agents’, ‘interaction’, ‘unpredictability’, ‘emergence’ and ‘context dependency’. We present definitions of these terms used for this analysis in Table 1.

**Table 1:** Definitions of complex adaptive systems concepts used for analysis

Complex adaptive systems terminology	Definition for analysis
Complex adaptive systems	‘At its core, a *complex adaptive system* comprises a population of diverse rules-based agents, located in multi-level and inter-connected systems in a network shape. A system is characterised by the behaviour of individual agents. Agents in complex adaptive systems are often numerous, dynamic, autonomous, highly interactive, learning and adaptive. Agents of complex adaptive systems act in ways that are based on a combination of their knowledge, experience, feedback from the environment, local values and formal system rules. These change over time leading to continuously changing interactions and adaptations that are often novel and are hard to predict, especially in social systems. Agents in complex adaptive systems interact with and adapt to each other and the system within the network. Complex adaptive systems are open systems with fuzzy boundaries and also highly context dependent in terms of time, history, and space including location and proximity. Complex adaptive systems also have distributed control. Consequently, complexity that is not necessarily a characteristic of individual agents, emerges at system level’ (Keshavarz *et al.*, 2010).
Diverse and dynamic agents	Agents ‘act in ways that are based on a combination of their knowledge, experience, feedback from the environment, local values and formal system rules’ (Keshavarz *et al.*, 2010). For this analysis, we define *diverse and dynamic agents* as intervention participants, facilitators and teachers.
Interaction	We consider *interaction* to be the interpersonal and group dynamics among these agents as well as between them and their family members or peers.
Unpredictability	*Unpredictability* is explained as continuous changes and adaptations in the system that may be ‘novel and are hard to predict, especially in social systems’ (Keshavarz *et al.*, 2010).
Emergence	We use the term *emergence* to reflect the unpredictable changes or outcomes in the system resulting from the ‘interplay of the many factors indicated above over time’ (Keshavarz *et al.*, 2010).
Context dependency	*Context dependency* suggests that individual agents will behave differently depending on the unique context within each system and that ‘different contexts create different influences on the way in which agents can function and on the complexity of introducing change’ (Keshavarz *et al.*, 2010). This concept is similar to that of *path dependence*, which considers systems as ‘sensitive to their initial conditions, so that the same force might affect seemingly similar organizations [systems] differently based on their histories’ (Lindberg and Schneider, 2013).

### Study design

We conducted a *post hoc* analysis and examined existing qualitative data from a case study evaluation against key attributes of complex adaptive systems. We designed this case study with a dual purpose: first, as an evaluation of a specific intervention in Mexico and, second, as a methodological exploration to apply and test different research methods, approaches and techniques during the course of the evaluation itself. The complex adaptive systems analysis presented in this article responds to this second purpose.

We conducted the evaluation study in one school in the south of Mexico City in 2017 and 2018. The aim was to learn about participant experiences in the intervention and explore whether and how it contributed to IPV prevention. Three partner organizations collaborated on study design and implementation: Fundación Mexicana para la Planeación Familiar, A.C. (Mexfam)—a Mexican non-governmental organization providing community-based health promotion as well as clinical services; International Planned Parenthood Federation/Western Hemisphere Region (IPPF/WHR)—an international non-governmental organization; and London School of Hygiene and Tropical Medicine (LSHTM). The first author was affiliated with the latter two at the time of the study and coordinated the collaboration.

The evaluation employed a longitudinal quasi-experimental design with an intervention and comparison group. Mixed methods of data collection were in-depth interviews, repeat (longitudinal) interviews, self-administered questionnaires, focus groups and observation. We collected data from students aged 14–17 years, teachers and Mexfam facilitators. Participants received a gift card for each interview or focus group as compensation for their time and were offered subsidized services at Mexfam clinics. We obtained ethical approval in Mexico from ‘Comité de Bioética y Ciencia para la Investigación, Centro de Investigación Clínica Acelerada’ (CICA) and in the UK from the LSHTM Research Ethics Committee. We have reported elsewhere on the intervention content and context, participant characteristics and evaluation findings—which suggest that the course promoted critical reflection that appeared to lead to changes in beliefs, intentions and behaviours related to gender, sexuality and relationships that supported the prevention of and response to IPV among young people (Makleff *et al.*, 2020).

The evaluated programme comprised 10 2-hour sessions delivered over one semester to mixed-gender groups of approximately 20 young people, who remained together for the duration of the course (the ‘intervention group’). The intervention was implemented with two groups of students in their third year of secondary school (*preparatoria* in Mexico) during the pilot phase and six groups in their first year of secondary school during the full implementation; there was a possibility of exchange between members of different intervention groups in their other courses. Each group was assigned a young (under 30) facilitator who was staff at Mexfam, where they were trained on the comprehensive sexuality education curriculum to ensure consistency in implementation while allowing for flexibility to adapt to emerging situations. The manual-based curriculum, designed based on international standards (United Nations Educational Scientific and Cultural Organization, 2018), comprised participatory activities, each building on prior sessions, to reinforce key messages about course topics. These included sexuality, gender, equitable relationships, IPV and other sexual and reproductive health topics. Core messages included the importance of self-respect, promoting tolerance of difference and considerations of power. IPV was presented as a range of behaviours that anyone in a relationship could perpetrate or experience, including excessive jealousy or control over a partner and other behaviours that cause emotional, physical or sexual harm. Activities were designed for factual learning, to encourage access to health services, to stimulate debate, reflection and group discussion and to build interpersonal, communication and relationship skills. The intervention was gender transformative in its aim to generate critical reflection about gendered social norms and shift individual attitudes and group norms related to gender, sexuality and violence.

### Data collection

Focus groups with course facilitators were conducted at Mexfam’s offices and co-facilitated by the research coordinator (J.G.) and the first author (S.M.)—both women. The objectives were to learn about intervention processes (challenges, group dynamics, activities) and facilitators’ perceptions of the course effects. All Mexfam facilitators who implemented the curriculum as part of the study, as well as the manager of the comprehensive sexuality education programme, were invited to participate. We conducted one focus group in June 2017 with the two facilitators (one woman and one man) who implemented the intervention pilot. We held another in December 2017 with the programme manager (female) and all four facilitators (three women, one man) who implemented the course during the full study. Two facilitators attended both focus groups. Participants had a mean age of 26.4 years (range: 23–29 years).

Nine students (five women and four men) with a mean age of 15.1 years participated in repeat semi-structured interviews over a 6-month period, during and after the intervention. We conducted 33 interviews in total; seven participants completed four interviews, one completed three and another completed two. The research coordinator (J.G.) conducted these either in a private space at the school or at Mexfam’s offices. Interviews included questions about experiences in and perceptions of the intervention, group dynamics and course effects. The sampling strategy and methodology of the repeat interviews are detailed elsewhere (Makleff *et al.*, 2020). Table 2 presents information about the facilitators and participants in each implementation group. We use pseudonyms to protect participant confidentiality.

**Table 2:** Characteristics of each intervention group

Group	Timeframe	Facilitator^a^	Gender balance	Repeat interview participants^a^	Summary of dynamics and events in each group
1	January–June 2017 (pilot)	Paola (F) with support from Orlando (M)	60% women 40% men	Laura (F) Gilberto (M)	Developed trust over time Learned to engage in respectful debate
2	January–June 2017 (pilot)	Regina (F) with support from Orlando (M)	80% women 20% men	None	Women in the group debated whether a female classmate was experiencing subtle forms of IPV. The classmate and her boyfriend were there and denied that their relationship was violent
3	August–December 2017	Patricia (F), then switched to Orlando (M)	55% women 45% men	None	Earthquake during session Change in facilitator after the earthquake was hard for participants to adapt to
4	August–December 2017	Berenice (F)	55% women 45% men	Beatriz (F) Elena (F) Israel (M) Julian (M)	Earthquake during session Conflict resolution session related to verbal aggression among participants Participant yelled at facilitator and hit classroom wall Some participants treated intervention as joke, distracted others Respectful dialogue regarding sexual diversity Male participants unwilling to participate in IPV-related activities
5	August–December 2017	Tania (F)	55% women 45% men	Karina (F) Lizbeth (F)	Negative comment about abortion triggered strong reaction among female participants and facilitator Some treated intervention as joke, distracted others Improvements in group dynamics over time Women took course more seriously than men
6	August–December 2017	Berenice (F)	55% women 45% men	Gerardo (M)	Some treated intervention as joke, distracted others Active engagement in activities, particular when group leader was present
7	August–December 2017	Regina (F)	55% women 45% men	None	Two men made aggressive comments about women; women in the class appeared to participate less as a result
8	August–December 2017	Orlando (M)	55% women 45% men	None	Women more interested in the IPV-related contents than men

a All names are pseudonyms.

### Analysis

Others have proposed that a qualitative approach is particularly fitting when applying complexity theory (Gear *et al.*, 2018; Gomersall, 2018). We reviewed qualitative evaluation data—transcripts from two focus groups with course facilitators and 33 repeat interviews—to identify examples of key elements of complex adaptive systems: interactions between diverse and dynamic agents, unpredictability and emergent outcomes and context dependency. We particularly sought examples where facilitators and students described the same events, allowing the comparison of different perspectives. We also reviewed the transcripts for evidence of the intervention acting as disruptive to a system (Moore *et al.*, 2019). Specifically, the analysis aimed to identify ways in which this comprehensive sexuality education programme with a gender-transformative approach was potentially ‘disruptive’ to the formal and informal rules, such as social norms, that govern the system—in this case, the intervention group and its participants.

## Results

The findings presented here demonstrate an application of complex adaptive systems concepts to qualitative evaluation data. We provide examples of how this approach can shed light on the ways in which interpersonal interactions, group dynamics, the core messages of the course and context influenced the implementation and outcomes of this intervention.

### Interactions between diverse and dynamic agents

The comprehensive sexuality education course promoted interaction in the intervention group setting. For the purposes of this analysis, we defined these groups, rather than the school as a whole, as the system being examined. We applied the concept of ‘diverse and dynamic agents’ from complexity theory to mean the approximately 20 participants and one facilitator engaged together in each mixed-gender intervention group. There were eight such groups in the study, two in the pilot and six in the full study. There were slightly more women than men assigned to each group, with some variation (Table 2). We examined narratives from facilitators and students to identify examples of how interactions and group dynamics influenced experiences in different groups. They described both positive and negative interactions. Positive experiences they mentioned included respectful dialogue and learning about the beliefs and experiences of other participants, while negative ones ranged from verbally aggressive behaviour to classmates not paying attention or interrupting class.

One facilitator, Berenice, mentioned an episode of physical aggression in Group 4. Specifically, she said that a female participant yelled at her and hit the wall before leaving in the middle of a session. Some participants also mentioned this event. In addition, Berenice and various students described bullying in the form of repeated teasing or mockery. Beatriz and Elena, students in this group, each mentioned that they had been bullied by classmates during the course. Beatriz spoke about her experience:

Sometimes my group bothers me a lot because there is a group of girls […]. Not just with me, with many of my classmates, they have hurt them with the things they say. […] They discriminate against me because of the colour of my skin. […] There are also kids in my class that they make feel bad, and they lower people’s self-esteem.

This example indicates that violence was exerted and experienced by both women and men and that different types of violence were perpetrated during the intervention sessions. Veteran teachers in the school also told us that this group of students was particularly unmanageable, and the facilitator (Berenice) noted Group 4 as an outlier in terms of group dynamics.

The difference was very noticeable. That is, not all classrooms have the same levels of violence, but in this group […] the violence was very marked. […] [In one activity] the group started to [verbally] attack each other, and it was one corner of the room against the other […]. The students became more and more out of control.

Berenice (the facilitator) also told us that after two participants (one woman and one man) escalated their verbally aggressive behaviour towards each other, she organized an activity—a spontaneous session that was not part of the regular curriculum—in which they had to directly communicate about their conflicts. Several students in Group 4 also talked about a conflict resolution exercise that addressed the fighting in the group. According to Beatriz, who participated in this activity, this session helped the two participants improve their relationship. ‘They started talking and became friends, they even go out together, it helped them a lot. […] Yes, [the course] influenced them, a lot. […] [To have a] better relationship.’ Julián, also in this group, did not mention this particular event but told us that, while at first the participants did not get along, they started to become more open because of the course, show more respect in class and pay more attention.

Berenice similarly mentioned improvements in the behaviour of the two participants who were in conflict, as well as in the group dynamics overall. However, she still expressed concerns about the ongoing aggression and violence in Group 4.

I’m a little worried because I gained their trust […] – not completely, not 100%. But I’m, like, worried that now in the next semester, […] they’ll change classes, there will be more violence. Some girls commented that they were going to change schools because they didn’t like the atmosphere, I mean straight up they said, listen, it’s very violent here and I don’t want to be here. Maybe not [violence in romantic] relationships, but yes, other types of violence.

Other, less severe negative interactions were also mentioned by participants from multiple groups. For example, Beatriz and Elena in Group 4, Lizbeth in Group 5 and Gerardo in Group 6 mentioned that some of their classmates treated the intervention as a joke or did not pay attention during the sessions, creating a distraction for those students who did want to participate.

Participants in various groups also described positive aspects of group dynamics or improvements in interactions over time. For example, two participants from Group 1 (Laura and Gilberto) and their facilitator (Paola) each told us the group learned to accept each other’s differences and debate respectfully. As Gilberto said, ‘[Paola] built trust, a warmth between everyone that was very nice, so much that we became like a little family again.’ Karina similarly talked about the openness and sharing that developed over time in Group 5. Israel described Group 4 as respectful and open regarding sexual orientation, saying he valued the opportunity to hear what others had to say.

What I noticed is that there is a lot of, like, diversity. It’s more that they don’t follow too much, um, this rule that boys like girls and girls like boys […] [A] girl […] said [to her friend], seriously, ‘Don’t you like Pedrito? […] Or, do you like Ana?’ And I noticed that and turned around to look. […] They do respect these types of things, and it’s, well, it’s something nice. […] In this class I’m like, twice as interested in seeing what the others will say, what we will talk about.

Using a complex adaptive systems approach brought attention to both positive and negative interpersonal interactions and how they influenced not only individual experiences but also the group-level dynamics in different implementation groups.

### Unpredictability and emergent outcomes

A complex adaptive systems approach anticipates continuous change in ways that are unpredictable and influence the system’s collective properties over time. We analysed the data to find reference to such unpredictable events or circumstances and present here three examples and their influence on collective experiences in the intervention.

The first example relates to how aggressive commentary in class can influence the group dynamic. In one case, the Group 5 facilitator (Tania) told us that a young man in class said was fine for a woman to die from an unsafe abortion, upsetting her as well as several young women in the class. Karina, a student in this group, said:

I don’t remember [the comment]. I think it was about the responsibility of having a baby, I don’t know, that the women had to stay home to take care of the baby, something like that, but [it] sounded very… bad, from my way of thinking.

Multiple participants told us that this comment was a significant event in Group 5, triggering a debate. Karina and Lizbeth each spoke about how female participants challenged the young man’s comment. They also said that they observed a gradual shift in the types of comments made by this young man afterwards. For example, several months after the intervention ended, Lizbeth said that she thought he was ‘no longer *machista* [male chauvinist]’ and had stopped making such comments.

Tania (the Group 5 facilitator) described this event, noting that young women in the group spoke out and also saying she perceived changes in this young man over time.

In the first session […] about unplanned pregnancy […] [one participant] made it be known that he had no problem with unsafe abortion […]. He said ‘well, she should die’ […]. And so, the girls got angry. […] And he said it again, like, very seriously […]. And from there I confronted him, and […] we started to question what he had said. And at the end of the session […] it seems he realized that his comment was really aggressive. […] Since then, he started to have more measured participation, also calmer […]. This change in, um, attitude and thoughts doesn’t happen in one hour, but […] he started with an extreme comment and then after that his comments became very, very mellow. And, like, throughout the sessions […] he ended up being one of those who participated the most, like when [the conversation] required reflection.

This provides an example of how female participants and the facilitator responded to an aggressive and gendered comment about abortion and appeared to disrupt a pattern of behaviour—in this case, gender-discriminatory commentary—to potentially shift the rules in the group about the acceptability of this type of commentary.

In Group 7, the facilitator (Regina) told us about two men in the group repeatedly making aggressive comments that reinforced harmful gender norms, such as blaming women for not getting contraception, wearing short skirts or having too many boyfriends. She said that, despite her attempts to address the situation, the men continued to make such comments. Over time, women in this group appeared to participate less when these men were present.

A second example of a circumstance that influenced the collective experience in the intervention had to do with the willingness of participants to openly share their personal experiences with the group. Some participants noted that gay or bisexual participants in their group spoke openly about their experiences during the intervention sessions. For example, Julián (Group 4) said he had never had a gay friend before and that through the course he initiated conversations with an openly gay classmate to ask questions about sexuality. He also said that the course helped participants learn about and accept sexual diversity, ‘to open their minds, you could say’. Israel, also in Group 4, identified himself as gay. He described these conversations from his own perspective, describing a male classmate who asked him many interesting questions about the lesbian, gay and bisexual community. Israel said that many of his classmates seemed genuinely interested in the topic of sexual diversity. It seems that the intervention methodology created a space in which participants could express themselves freely about sexual orientation and influence the beliefs and social norms in the group around sexual diversity.

A third unpredictable event that influenced the collective experience was a magnitude 7.1 earthquake that hit central Mexico on 19 September 2017. This earthquake took place a few weeks after the intervention began, during concurrent sessions of the intervention attended by Groups 3 and 4. Participants, facilitators and teachers reported that the event was traumatic for many of them. Afterwards, Mexico City schools were closed until the buildings could be inspected. Participants, facilitators and teachers did not know when the school would reopen. Ultimately, the semester restarted after 3 weeks and the intervention continued in a slightly condensed form. The Group 3 facilitator (Patricia) left her position at Mexfam after the earthquake and was replaced by Orlando. Orlando and some participants told us that it was difficult for the group to readjust to a new facilitator in the middle of the course. We do not know the direct influence of the earthquake on intervention outcomes, but the facilitators told us that after weeks of school closures they had to rebuild group trust and address the emotions and trauma related to the earthquake. This further delayed implementation, reducing the hours available for the curriculum.

### Context dependency

A complex adaptive systems approach expects context dependency, meaning that different participants (sub-systems) and intervention groups (systems) will respond differently to the intervention based on their initial conditions and context. We found a striking example of this, in which there were different responses to IPV-focused activities in two intervention groups that had the same facilitator. According to this facilitator, Berenice, the young men in Group 4 were unwilling to engage in the topic of IPV.

I noted that the girls were much more interested than the boys […]. The boys […] told me, ‘I’m not interested at all in violence’ […]. And the girls were the ones who went and started to debate with the boys about why it was important for them to learn about this, no? And the boys said ‘it’s that really, I’m not interested, and you can tell me a thousand and one arguments, and no.’ And in the last sessions I noticed more attendance by the girls than the boys.

Group 6 experienced the same curriculum as Group 4, also facilitated by Berenice, yet responded differently to the IPV-related activities. In Group 6, an influential young man was enthusiastic about the intervention and seemed to encourage classmates to engage actively, particularly on the topic of IPV. Berenice said:

The head of the group went on vacation, and he is a born leader, because when he went on vacation many of them stopped coming. And, when [he] came back, they came back, no? […]. And when we started to talk about violence, the group leader was the one who began to participate the most, the one who showed most interest, and [following his lead], all the rest seemed much more interested in what we had to say. There was a moment when I didn’t even talk. That is, I was just listening, and they were sharing. And, well, it was a circle of trust, they all started to share their ideas.

Another example of a possible gender difference in responses to the course was mentioned by the Group 8 facilitator, Orlando, who told us that women took course more seriously than men. Similarly, Lizbeth, a participant in Group 5, told us that the women in her group took the course more seriously than the men:

The men take these things as a joke, that’s why sometimes there are unwanted pregnancies because they don’t take it seriously. [Instead of saying,] ‘well, if my girlfriend doesn’t know something, I’ll pay attention so I can talk about it [with her],’ it’s like ‘Hahaha, condoms, oh hahaha, this and that.’ […] I feel like the girls are taking it more seriously.

These narratives may indicate a gendered difference in how participants respond to the course, depending on the dominant gender norms and other power dynamics within each of the groups.

### System-disruptive elements of the intervention

The comprehensive sexuality education programme being evaluated was designed with a gender-transformative approach, seeking to reshape pervasive gender norms within a system. Therefore, we examined the focus group and repeat interview data to assess whether there was evidence that the intervention disrupted norms within the intervention group or influenced gender- and IPV-related beliefs among participants.

Multiple participants described their perception that the intervention introduced new ideas that countered the status quo in terms of common beliefs and norms about relationships and IPV. Key intervention messages mentioned by participants were: (1) respecting oneself and one’s own needs in a relationship, (2) how to behave in a relationship and the many types of IPV than can occur and escalate and (3) the value of expressing yourself freely and accepting diversity, particularly regarding gender and sexuality.

The first core message about self-respect was mentioned by several women; none of the men directly mentioned this message, though several did describe learning to accept or respect their own preferences and needs in a relationship. The concept of self-respect seems to have influenced perceptions of how one should behave and expect to be treated in a relationship. Reflecting on this message, Lizbeth (Group 5) told us:

The most important part was this, to love myself. And that if I say something, it will be respected, because I’m saying it […]. Maybe others don’t respect it, but I myself will respect my own decision. […] If someone else don’t respect me, it’s enough that I respect my decision.

Lizbeth said that the course taught her to stand by her own decisions, changed her expectations of how her boyfriend should treat her and helped her behave differently within that relationship. For example, she said:

It used to be like, ‘it doesn’t matter if you go out with another girl, we’ll have sex anyway.’ And now it is like, ‘you want to be with someone else? Then leave.’ […] My character has become more… I do something because I said it.

The second core message, about the types of behaviour that are considered acceptable in a relationship, seems to have shifted perceptions among male and female participants. For example, Israel (Group 4) said that the course helped ‘differentiate between what [type of behaviour] is love and what is not love’. In another example, Laura (Group 1) contrasted what she learned about relationships in the course to the beliefs and norms in her family, demonstrating the potentially disruptive influence of the course on her own understanding of acceptable behaviour in relationships despite what she learned at home.

In my mother’s family, violence is that someone hits you. But they don’t know that violence can also be that someone insults you, doesn’t give you money […]. Because I’m sure that none of my aunts, nor my grandmother, are aware that violence is to pinch them, to push them, to be verbally insulted, to not receive money to buy food or to survive, or that type of thing. That is what I liked the most [about the course].

The third core message described by participants was the importance of respecting diversity and expressing yourself freely, particularly regarding gender and sexuality. This was mentioned particularly by participants who identified as gay or bisexual. Gerardo (Group 6) said the course taught ‘that you don’t need to comply with stereotypes, the labels that people place on you. Like, if you are a man you have to like women and if you are a woman you have to like men. So, well, I feel that it [the course] gives you a basis to respect people’s rights’. Israel (Group 4) similarly talked about the message that people should act freely and be themselves. He described an activity that caught his attention in the first intervention session, where the facilitator (Berenice) began to ask why the school uniform did not include trousers for women or skirts for men and why men should not wear pink and women should not wear blue.

I understood that the objective of the class was that we can express ourselves just as we are and don’t need to be guided by what people say about ‘boys should play with that, and girls should play that’.

Gilberto and Laura (Group 1) each talked about the process of learning to respect differences in opinion and debate respectfully. For example, Gilberto described a debate about social norms in the group:

We were all debating our points of view, arguing amongst ourselves but respecting each other. So we always were able to understand, and always respected everyone, and more than anything, sharing our opinion. And at best these did differ […] and we made comparisons about social stereotypes that we have from childhood, the strong social stereotypes about how to be a woman, how to be a man.

These core messages, which were part of a gender-transformative approach, positioned the intervention to influence and shift the gendered social norms and related beliefs that dictate how people should behave towards others, engage in relationships and otherwise express themselves.

## Discussion

The conceptualization of an intervention as a disruptive event in a complex system (Moore *et al.*, 2019) may be particularly well-suited for evaluating gender-transformative programmes. Such interventions aim to reshape—or disrupt—gender norms within local contexts. Applying the lens of system disruption to qualitative evaluation data helped us identify the key aspects of a comprehensive sexuality education programme in Mexico that appeared to disrupt the formal and informal rules that govern individual and group behaviours and beliefs, particularly those related to gender, relationships and IPV. The specific messages in Mexfam’s intervention about self-respect, how to treat others and acceptance of diversity may have appeal beyond this particular context because they are broadly applicable regardless of location, gender, sexual orientation or relationship status. These messages seek to disrupt the rules of interaction in the system (the implementation group), including the gender system in that group—i.e. what is expected of women and of men (Hirdman, 1991; Heise *et al.*, 2019). Because gender norms uphold and underlie gender systems (Heise *et al.*, 2019), gender-transformative programmes aiming to shift these norms are ultimately attempting to reshape the gender system in a community to be more equitable. By employing a complex systems approach, we were able to connect examples of individual-level changes or actions in the group setting to potential system-level shifts in the intervention group—as in the case of female participants speaking out against a classmate’s gender-discriminatory comment about abortion to influence what was considered appropriate to say in the group.

Our analysis shows that without examining both individual and group processes, evaluations may miss key insights about how the intervention generates change, for whom and how it interacts with its context. Complementing the individual analyses so common in evaluation practice with a complex adaptive systems approach can help ‘see the wood as well as the trees’ when conducting public health evaluation, as advocated by Smith and Petticrew (2010). This combination of approaches allowed us to better understand the variability we observed in implementation environments, actors and outcomes.

A systems approach to evaluation acknowledges that social interventions are unpredictable and emergent. As noted in the UK Medical Research Council guidance for evaluating complex interventions, fidelity in implementing these types of interventions ‘is not straightforward’ (Craig *et al.*, 2008). Therefore, it may not be reasonable to expect standardized implementation processes and consistent outcomes. In our study, we identified two similar groups of students who received the same intervention from the same facilitator, but their responses varied dramatically. Similarly, a vignette-based activity was implemented in all implementation groups, but only in one group did it seem to trigger a heated debate about gender norms and eventual shift in the conduct of some participants. These examples highlight the potential for variable responses to an intervention, even when implemented concurrently and in the same setting. In addition to the ways in which social interactions can lead to variation, a range of external factors can also contribute to variability in implementation. In our study, an earthquake impeded our ability to implement the intervention as planned. Such experiences raise questions about whether fidelity is a relevant concept (Hawe *et al.*, 2004; 2009) and challenge the concept of faithful implementation when implementing social interventions embedded in complex systems—which are context dependent, behave unpredictably and have emergent results that develop over time.

A complex adaptive systems approach to analysis helped us assess how unanticipated events such as the earthquake or particularly aggressive group dynamics seemed to influence individual participants as well as collective experiences. The experience in Mexico suggests that, with well-trained and supported facilitators and a flexible curriculum, implementing teams can cope with unpredictable events and suboptimal implementation environments to constructively address implementation challenges and contribute to IPV prevention efforts. Flexibility to respond to challenges and adapt to local needs is important when implementing comprehensive sexuality education; a study of these programmes in four low- and middle-income countries highlighted the need for ‘mechanisms for feedback on implementation hurdles’ and adaptation of curricula for different contexts (Keogh *et al.*, 2018).

A systems analysis can help organizations prepare for different types of unpredictable occurrences and outcomes that may emerge during an intervention (Peters, 2014), in part by putting a spotlight on some of the most challenging aspects of implementation. For example, the intervention in Mexico was promoting equity, respect and non-violent relationships while also, unintentionally and unwillingly, serving as a space in which bullying and aggression were perpetrated. Bullying is a common form of school violence and can entail physical, psychological or sexual forms of aggression (United Nations Educational Scientific and Cultural Organization, 2019). This highlights a tension inherent in implementing school-based violence-prevention interventions: because of the ongoing violence that can permeate schools, it may not be possible to implement school-based interventions in truly violence-free spaces. This raises questions about how interventions seeking to prevent IPV or other forms of violence can reduce the potential for harm and help avert normalized forms of interpersonal violence—a concern also noted in a study of an IPV prevention programme in South Africa (Hatcher *et al.*, 2020). As good practice, training in classroom management and conflict resolution should equip facilitators to address bullying, aggressive behaviour and other forms of harmful group dynamics. In the case study presented in this article, facilitators were trained in these topics and did respond directly to aggressive behaviour in the course. Despite this, interpersonal conflicts had a negative influence on the experiences of other participants. Although Mexfam routinely trains its facilitators in classroom management, based on this experience the organization is developing a set of tools to systematically support their staff in addressing any harmful or aggressive behaviour that occurs during intervention implementation. Similar tools may be useful to support facilitators of school-based programmes addressing a range of types of violence in different contexts.

Despite this preparation and the programme’s gender-transformative approach, and although the evaluation results suggested that the intervention in Mexico had positive effects on both young men and young women (Makleff *et al.*, 2020), we observed some differences in responses to the intervention by gender. Overall, women appeared to take the course more seriously than men, though many men did engage actively during the course—in particular in one group with a socially influential male participant who encouraged classmates to participate. Several facilitators encountered varying degrees of resistance to the course among male participants, especially related to the topic of IPV. These findings have programmatic implications, highlighting the importance of working with both men and women while also identifying further strategies that effectively engage men in IPV prevention programming—also noted by other researchers (Peacock and Barker, 2014; Dworkin and Barker, 2019). Other IPV prevention programmes have similarly found that male participants sometimes rebelled against or resisted intervention messages (McGeeney, 2015; Pierotti *et al.*, 2018). A study in South Africa described ways in which male facilitators of an IPV prevention intervention negotiated and engaged their own masculinities—sometimes limiting the potential of the programme to contribute to transformational change (Gibbs *et al.*, 2019). These examples are in line with the finding from a recent systematic review that gender-based violence interventions appear more effective at reducing exposure to IPV among young women than at reducing exposure or perpetration among young men (Meinck *et al.*, 2019).

This study had a number of limitations. First, the study was not designed with a complex adaptive systems perspective specifically in mind and the analysis was conducted *post hoc* to complement individual-level analyses published elsewhere. However, the study design did include methodological exploration to take into account dynamic and contextual aspects of the intervention, which inspired the complex adaptive systems approach presented here. Second, the few programme activities that might influence school-level change, such as teacher training and a health fair, were implemented after the study ended to avoid contamination of the evaluation. Changes at the school or school system level would be relevant to examine from a systems perspective in future studies. Third, we included in this article only a subset of complexity concepts, chosen for their relevance to these particular data. Because this article aimed to explore the relevance and potential added value of a complex adaptive systems approach to evaluation, our intention was not to conduct a systematic assessment of all of the data from the evaluation in Mexico using all possible complexity-related concepts. In addition, complexity theory and complex adaptive systems are inconsistently defined and applied across studies (Walton, 2014; Thompson *et al.*, 2016), exacerbating challenges in defining an appropriate set of concepts that should be included in such an analysis. Future evaluation studies could contribute to refining the set of complexity concepts most relevant to intervention evaluation and consider adopting a systems approach to complement other analyses. This may be particularly relevant when evaluating gender-transformative programming and interventions that address complex social issues such as IPV. Finally, it would be interesting to engage a complexity approach to examine how system-level changes influenced by the intervention are sustained—or not—over time, but we do not have the long-term data needed to conduct this analysis.

## Conclusion

An intervention is a composite of multiple dynamic and interacting elements beyond the (somewhat static) curriculum, including each participant’s background and experiences, facilitator characteristics, group dynamics and environmental or contextual factors—all of which are unlikely to be consistent across implementation settings. Applying complexity concepts to our analysis helped us reframe implementation-related data to focus on how the ‘social’ aspects of complexity, particularly interactions among participants and facilitators, influenced the intervention. A complex adaptive systems approach also sheds light on some of the variation in experiences and outcomes across individuals and groups. A system-level focus is a useful complement to individual-level analyses, which may fall short when examining complex and norms-based outcomes such as IPV. A social complex adaptive systems approach is well-suited to the evaluation of gender-transformative interventions and can help identify how such interventions disrupt the complex social systems in which they are implemented to address intractable societal problems.
